# Genetic Deletion of Prostacyclin IP Receptor Exacerbates Transient Global Cerebral Ischemia in Aging Mice

**DOI:** 10.3390/brainsci3031095

**Published:** 2013-07-22

**Authors:** Hania Shakil, Sofiyan Saleem

**Affiliations:** 1 Hamdard College of Medicine and Dentistry, Hamdard University, Sharae Madinat Al-Hikmah, Karachi 74600, Pakistan; 2 Center for Neuroscience, Aging and Stem Cell Research, Sanford Burnham Medical Research Institute, La Jolla, CA 92037, USA

**Keywords:** global cerebral ischemia, aging, cognitive deficit, prostaglandin I2, mouse

## Abstract

Transient global cerebral ischemia causes delayed neuronal death in the hippocampal CA1 region. It also induces an up regulation of cyclooxygenase 2 (COX-2), which generates several metabolites of arachidonic acid, known as prostanoids, including Prostaglandin I_2_ (PGI_2_). The present study investigated whether the PGI2 IP receptor plays an important role in brain injury after global cerebral ischemia in aged mice. Adult young (2–3 months) and aged (12–15 months) male C57Bl/6 wild-type (WT) or IP receptor knockout (IP KO) mice underwent a 12 min bilateral common carotid artery occlusion (BCCAO) or a sham surgery. Behavior tests (neurologic deficit and T-maze) were performed 3 and 7 days after BCCAO. After seven days of reperfusion, the numbers of cells positive for markers of neurons, astrocytes, microglia, myeloperoxidase (MPO) and phosphorylated CREB (p-CREB) were evaluated immunohistochemically. Interestingly, in young and aged IP KO ischemic mice, there was a significant increase (*p* < 0.01) in cognitive deficit, hippocampal CA1 pyramidal neuron death, microglia and MPO activation, while p-CREB was reduced as compared to their corresponding WT controls. These data suggest that following ischemia, IP receptor deletion contributes to memory and cognitive deficits regulated by the CREB pathway and that treatment with IP receptor agonists could be a useful target to prevent harmful consequences.

## 1. Introduction

Transient global cerebral ischemia, arising in humans, can be an aftermath of cardiac arrest or severe systemic hypotension. It leads to major neuropsychological dysfunctions, including learning and memory disabilities especially in aged populations [1,2]. Researchers have struggled to discover an effective therapeutic approach because the pathophysiologic mechanism of forebrain ischemia/reperfusion injury is very complex, especially in the aged population [3]. For instance, one needs to take into account intracellular calcium (Ca^2+^) overload, the toxic effects of excitatory amino acids, an aberrant increase in oxygen free radicals, the activation of inflammatory factors and death gene regulation [4].

Recent reports suggest that both cyclooxygenase (COX)-1 and -2 play a crucial role in inflammation, but they have different functions [5]. COX-2 is highly inducible by inflammatory stimuli and increases the production of five prostanoids: prostaglandin PGE_2_, (PG) D_2_, PGF_2α_, PGI_2_, and thromboxane A_2_ [6]. These prostanoids mediate their effects mainly through G-protein-coupled receptors, *i.e.*, EP (1–4), DP (1–2), FP, IP and TP. Each of these receptors differs in their respective actions on cyclic AMP (cAMP), phosphatidyl inositol turnover, and intracellular Ca^2+^ mobilization that can lead to either protective or harmful effects [7].

Prostaglandin I2 (PGI2) is derived from sequential metabolism of arachidonic acid (AA) via cycloxygenase and prostaglandin I_2_ (PGI_2_) synthetase (PGIS), and acts mainly on the membrane-bound IP receptors [8]. It is an endogenous vasodilator and inhibitor of leukocyte adhesion and platelet aggregation [9]. PGI2 analogs have been reported to protect against ischemic stroke damage in gerbils and patients [10,11]. The PGI2 IP receptor has been reported to decrease traumatic brain injury and focal cerebral ischemia [12,13]. It has been reported that prostacyclin suppresses cardiac fibrosis by inducing phosphorylation of CREB [14], which is also involved in short- and long-term learning and memory [15,16].

In this study, we investigated the role of the IP receptor by measuring neurologic and cognitive deficits as well as neuronal death in young and aged WT and IP KO mice subjected to the BCCAO stroke model. In addition, we determined the degree of microglia activation and leukocyte infiltration as measured by MPO levels. We also quantified the phosphorylation of CREB, which interacts with the transcription co-activator CREB-binding protein to initiate the transcription and translation of CREB target genes, which are required for synaptic plasticity, learning and memory.

## 2. Results

### 2.1. No Change in Cerebral Blood Flow after Global Cerebral Ischemia in Young and Aged WT and IP KO Mice

A 12-min period of global ischemia induced a significant decrease in regional cerebral blood flow (rCBF) as measured by a laser Doppler flowmeter in young and aged C57Bl/6 WT (9.6% ± 0.6%; 9.0% ± 1.0% from baseline) and IP KO mice (8.8% ± 1.3%; 8.0% ± 1.0% from baseline) compared to corresponding sham-operated controls. There were no significant differences in the rCBF between the groups. The core body temperature was maintained between 37.0 and 37.5 °C in all mice.

### 2.2. Cognitive Deficiency and Motor Dysfunction Induced by Global Cerebral Ischemia Were Enhanced in Young and Aged IP KO Mice

**Figure 1:**
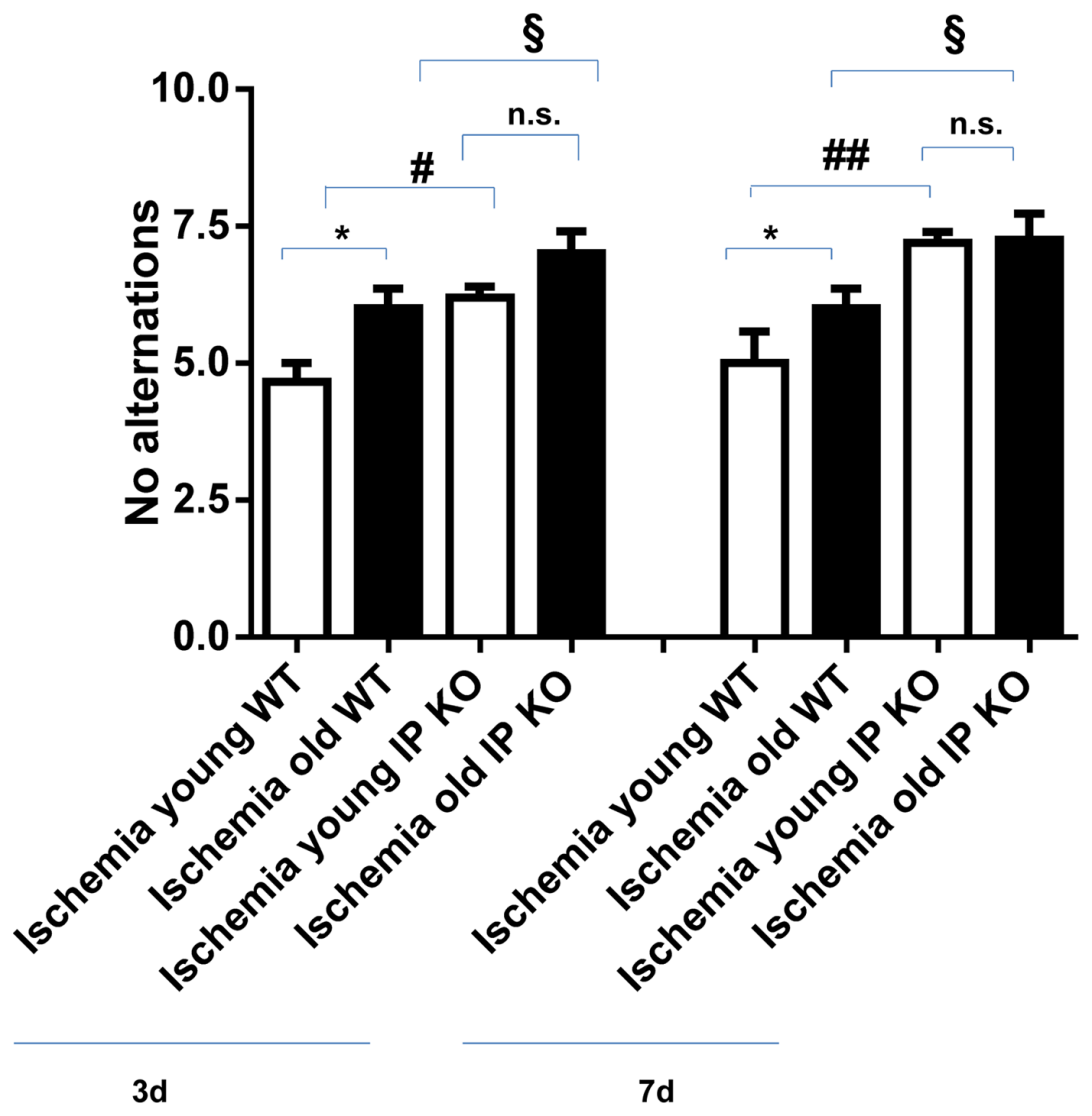
Number of “no alternations” in the T-maze spontaneous alternation test (10 trials) after 12 min global ischemia and 3-day or 7-day reperfusion. “No alternations” was assessed in both young and aged ischemic IP receptor knock out (IP KO) mice compared to their ischemic wild-type (WT) controls (* *p* < 0.05, ^§^ *p* < 0.05, ^#^ *p* < 0.05 and n.s. = no significance). Data represent mean ± s.e.m. *n* = 6–8 per group.

T-maze testing for spontaneous alternation demonstrated a significantly higher number of no alternations in the young (*p* < 0.05) and aged (*p* < 0.01) IP KO ischemia mice at day 3 compared to age-matched WT ischemia mice. By day 7, there were statistically significant differences in the young (*p* < 0.05) and aged IP KO (*p* < 0.01) ischemia groups as compared to age-matched ischemic WT controls (see Figure 1).

**Figure 2:**
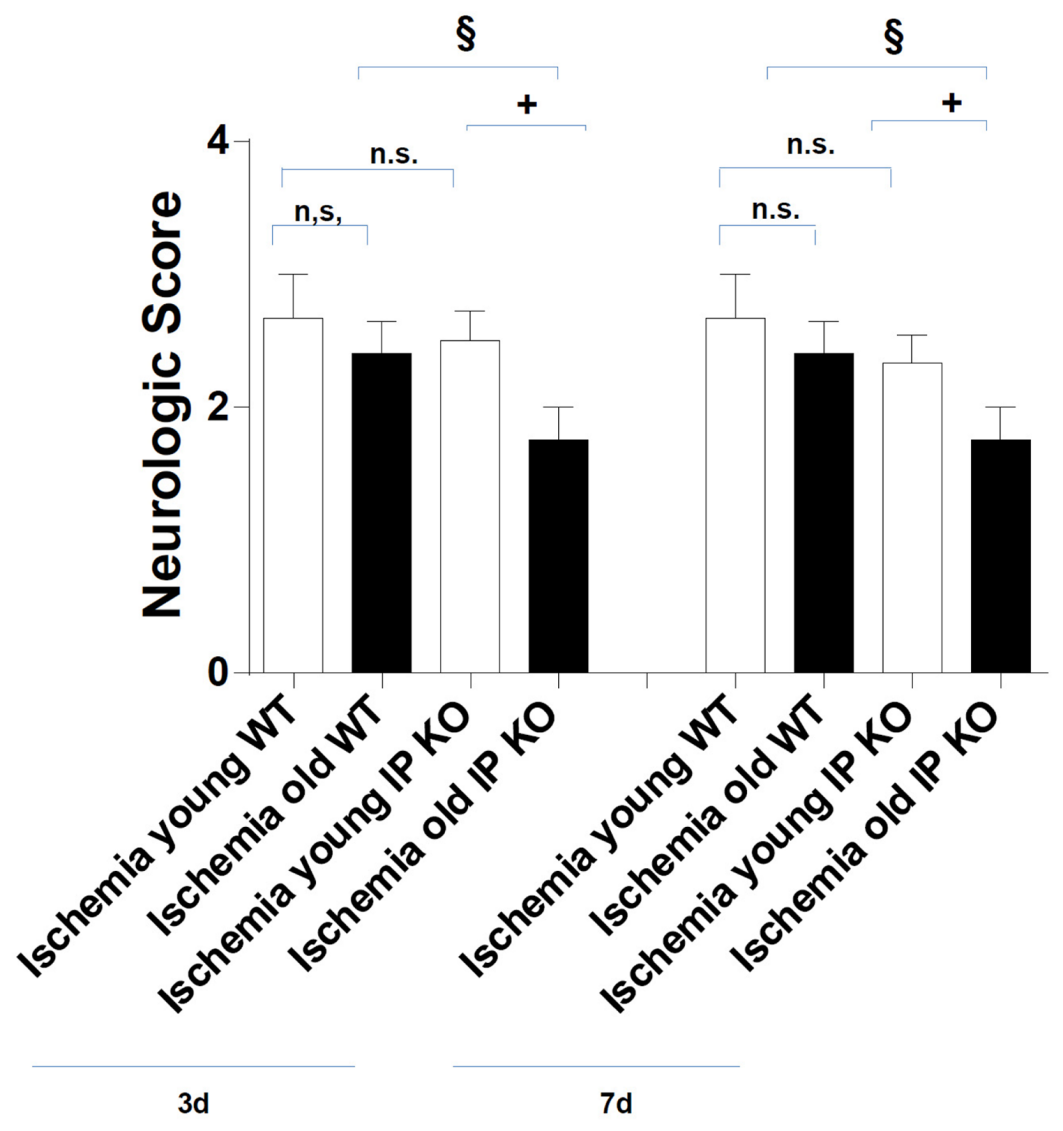
Effect of PGI2 IP receptor on locomotor activity after 12 min global ischemia and 3-day or 7-day reperfusion. Locomotor activity was assessed by a 5-point scale. Both young and aged ischemic IP KO mice were compared to their ischemic WT controls (^§^ *p* < 0.05, ^+^ *p* <0.05, n.s. = no significant). Data represent mean ± s.e.m. *n* = 6–8 per group.

For the field walking score at day 3, young IP KO mice (2.50 ± 0.22) showed no significance in uncoordinated spontaneous movement of groups of muscles as compared to young C57Bl/6 WT ischemic mice (2.67 ± 0.33). The mice in both groups showed movement of two or all three major joints in the hind limbs and active support and uncoordinated gait or short bouts of coordinated gait. On the other hand, aged IP KO mice (1.75 ± 0.25) showed more poorly coordinated movement (*p* < 0.01) as compared to the corresponding wild-type aged mice (2.40 ± 0.22), which showed uncoordinated spontaneous movement of groups of muscles. At day 7, the aged IP KO mice (1.75 ± 0.25) showed a more uncoordinated locomotor activity score (*p* < 0.01) compared to aged WT ischemic mice (2.40 ± 0.24) as shown in Figure 2.

### 2.3. Genetic Deletion of IP Receptor Enhanced Hippocampal CA1 Neuronal Cell Loss in Young and Aged IP KO Mice

**Figure 3:**
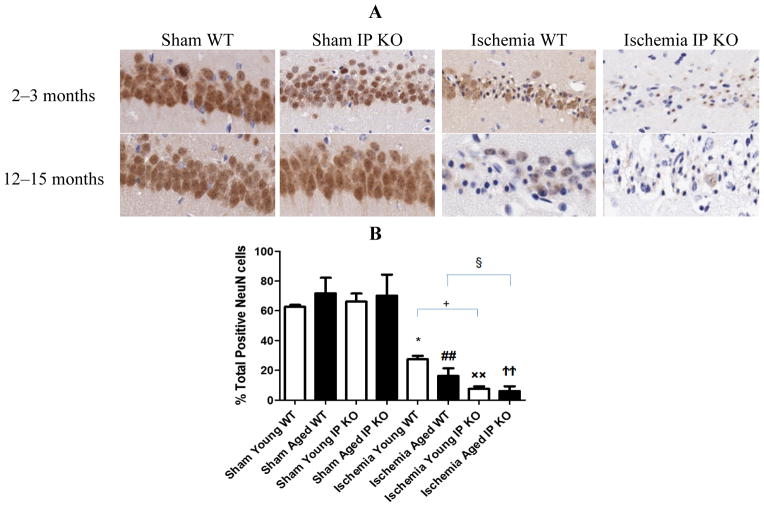
NeuN staining in young and aged WT and IP KO sham and ischemic mice after 12 min global cerebral ischemic insult. (**A**) Representative images of brain sections show NeuN immunostaining in young and aged WT and IP KO sham and ischemic mice. (**B**) Quantification of NeuN-positive cells in the hippocampal CA1 subfield on day 7 after ischemia, comparing ischemic IP KO groups with their sham and ischemic controls (*n* = 3 per group). * *p* < 0.05, ^××^ *p* < 0.01, ^##^ *p* < 0.01, ^††^*p* < 0.01 for Ischemia *vs.* Sham; ^+^ *p* < 0.05, ^§^ *p* < 0.05 for Ischemia IP KO *vs.* Ischemia WT.

**Figure 4:**
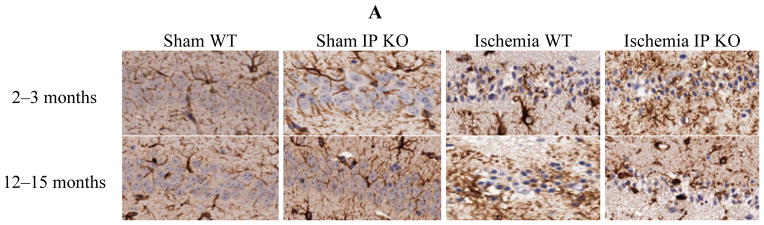

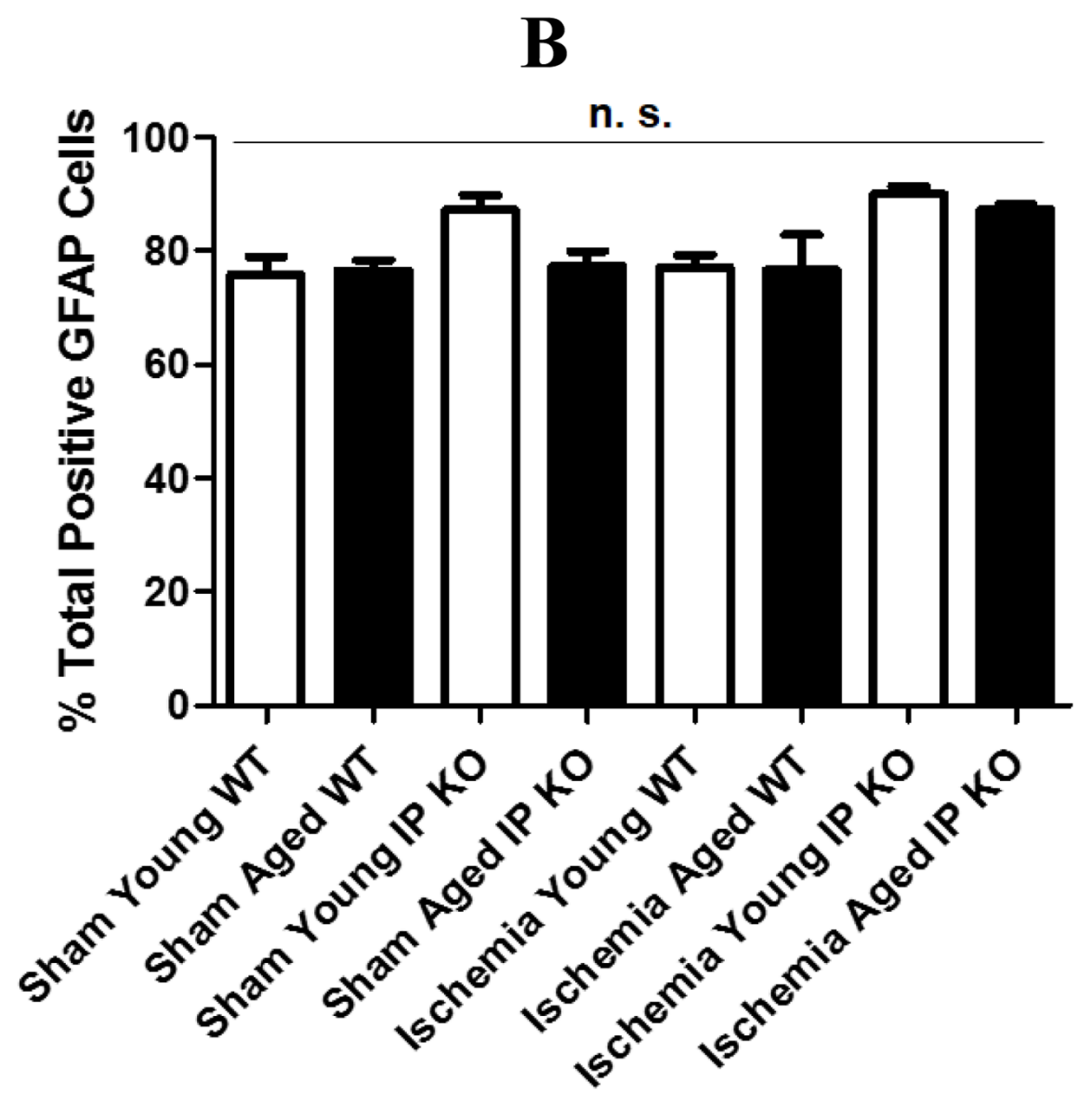
Astrocyte reactivity in young and aged WT and IP KO sham and ischemic mice after 12-min global cerebral ischemic insult. (**A**) Representative images of brain sections show astrocyte immunostaining. (**B**) Quantification of GFAP-positive cells in the hippocampal CA1 subfield on day 7 after ischemia (*n* = 3 per group). n.s. = no significance.

We then wished to correlate the behavioral phenotype with ischemic brain pathology, so we assessed delayed hippocampus neuronal damage following global ischemia. After seven days of recovery, the number of viable neurons in the hippocampus CA1 region was not significantly different among the sham groups of the various genotypes. However, the CA1 neuronal loss was much greater in the young and aged IP KO mice (*p* < 0.01) than in the control ischemic WT mice (Figure 3); no statistical differences were noted among young and aged WT and IP KO groups in GFAP (astrocyte marker) (Figure 4).

### 2.4. Genetic Deletion of IP Receptor Exacerbated Microglia Activation and Neutrophil Infiltration in Young and Aged IP KO Mice

**Figure 5:**
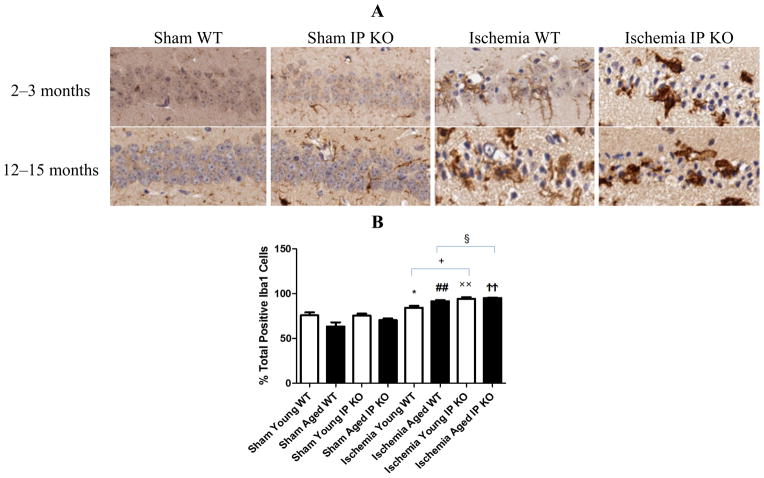
Microglia reactivity in young and aged WT and IP KO sham and ischemic mice after 12 min global cerebral ischemic insult. (**A**) Representative images of brain sections show microglia immunostaining. (**B**) Quantification of Iba-1-positive cells in the hippocampal CA1 subfield on day 7 after ischemia. (*n* = 3 per group). * *p* < 0.05, ^##^ *p* < 0.01, ^××^ *p* < 0.01, ^††^*p* < 0.01 for Ischemia *vs.* Sham; ^+^ *p* < 0.05, ^§^ *p* < 0.05 for Ischemia IP KO *vs.* Ischemia WT.

**Figure 6:**
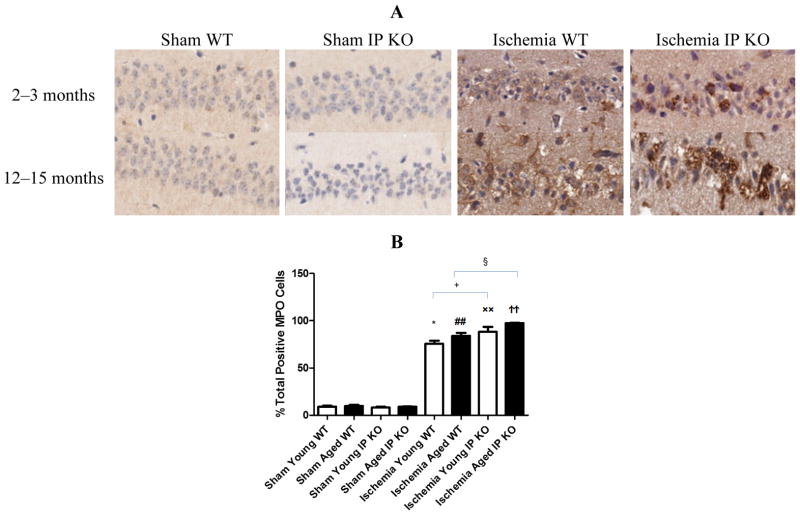
Myeloperoxidase activity in young and aged WT and IP KO ischemic mice after 12 min global cerebral ischemic insult. (**A**) Representative images of brain sections show MPO immunostaining. (**B**) Quantification of MPO positive cells in the hippocampal CA1 subfield on day 7 after ischemia. (*n* = 3 per group). * *p* < 0.05, ^##^ *p* < 0.01, ^××^ *p* < 0.01, ^††^*p* < 0.01 for Ischemia *vs.* Sham; ^+^ *p* < 0.05, ^§^ *p* < 0.05 for Ischemia IP KO *vs.* Ischemia WT.

Despite the lack of astroglial activation, microglial reactivity (Iba1-positive cells), and infiltrating neutrophils, measured as the level of MPO, in the hippocampal CA1 region after 12 min of global ischemia and seven days of reperfusion, was significantly elevated (*p* < 0.01) in young and aged IP KO ischemic mice compared to WT ischemic counterparts (*p* < 0.01) (Figures 5 and 6). These results indicate that IP receptor deletion exacerbates ischemia-induced neuronal damage resulting in activation of microglia and neutrophil infiltration in this mouse global ischemia model.

### 2.5. PGI2 IP Receptor Regulates CREB Phosphorylation after Global Cerebral Ischemia

**Figure 7:**
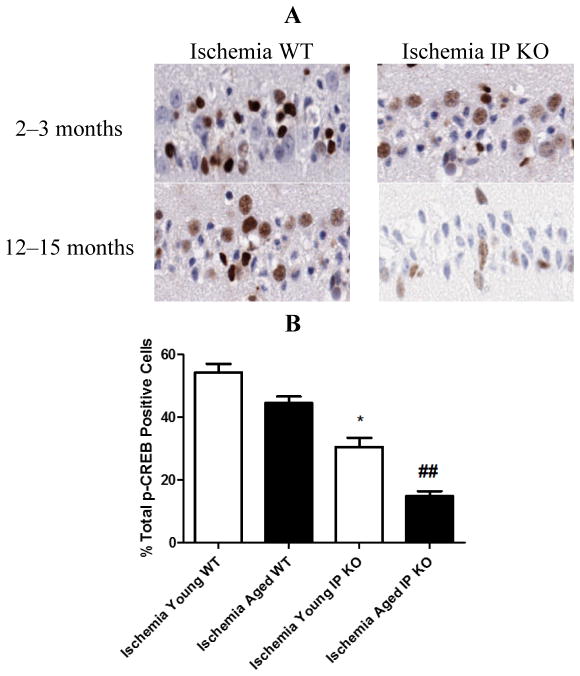
The phosphorylation of CREB in young and aged WT and IP KO mice after 12 min global cerebral ischemic insult. (**A**) Representative images of brain sections show p-CREB immunostaining. (**B**) Quantification of p-CREB positive cells in the hippocampal CA1 subfield on day 7 after ischemia. (*n* = 3 per group). * *p* < 0.05, ^##^ *p* < 0.01 for IP KO *vs.* WT.

In young and aged IP KO mice, the levels of phosphorylated CREB were significantly decreased (*p* < 0.01) as compared to the WT ischemic mice (Figure 7). However, there was no p-CREB immunostaining observed between young and aged WT and IP KO sham mice.

## 3. Discussion

The present study demonstrates the importance of PGI2 IP receptor in transient forebrain ischemia, especially in an aged population. Here, we showed that young and aged mice with a genetic deletion of the IP receptor have marked hippocampal injuries and memory impairments as evaluated by immunohistochemistry, and by the open field and T-maze tests as compared to their WT ischemic controls. This study correlates well with prior reports that suggest that PGI2 generated through COX-2 activity may play an ameliorative role in focal and global cerebral ischemia [10,13], and reduce motor disturbances and pathological damage following spinal cord and hypoxic injury in both adult and aged mice [17,18]. We now show that, some of these behavioral impairments and neuronal cell loss may be associated with the increase in the number of activated microglia and the reduction in CREB phosphorylation after genetic deletion of the IP receptor as compared to their corresponding WT ischemic mice.

Several reports claim that global cerebral ischemia, arising as a sequel of cardiac arrest or cardiac surgery in humans or induced experimentally in animals, leads to spatial cognitive impairments and hippocampal damage, especially in the CA1 region [19,20]. In the present study, we observed that young and aged IP KO mice with transient global ischemia by BCCAO exhibit serious motor and memory impairment, as determined by open field and T-maze tests, compared to WT ischemic mice. These results suggest that genetic deletion of the IP receptor exacerbates the memory dysfunction caused by BCCAO in young and aged mice by enhancing neuronal cell loss.

Increasing numbers of reports suggest that inflammation has been implicated as an important cause of the neuronal damage induced by ischemic conditions [21]. Microglia, a macrophage precursor in the brain, acts as a detector of inflammation and pathologic changes in the central nervous system. They rapidly become activated after brain ischemia or trauma [22]. It has also been reported that, although reperfusion following ischemia restores cerebral blood flow, it can lead to secondary brain damage from an influx of neutrophils and to increases in oxidative stress, brain edema, and hemorrhage [23]. In addition, myeloperoxidase (MPO), a key inflammatory enzyme secreted by activated neutrophils and macrophages/microglia, can generate highly reactive oxygen species to cause additional damage in brain ischemia [24]. Moreover, recent studies suggested that microglia activation suppresses the hippocampal-dependent spatial working memory in aged mice [21,25]. We found a significant increase in cognitive dysfunction and microglia activation and neutrophil infiltration after 12 min of ischemia and seven days of reperfusion in young and aged IP KO mice as compared to the WT ischemic mice.

It is well known that use of COX-2 inhibitors worsens heart disease and stroke [26]. Therefore, it is important to define the COX-2 mediated downstream pathways mediated through G-protein coupled receptors, such as the PGI2 IP receptor. Intervention in these pathways could prove beneficial against cognitive impairments and neuronal cell loss in aged populations [27]. It has been reported that activated IP receptors signal mainly through G_αs_ activation of adenylyl cyclase and mediates its neuro protective functions, mainly through the cAMP/protein kinase A signaling pathway [13]. Moreover, phosphorylation of CREB (p-CREB), the activated form of CREB, regulates many aspects of neuronal function, including short- and long-term memory formation [28–30]. Therefore, the activation of cAMP/PKA/CREB signaling pathway in the hippocampus should play an important role in spatial memory formation [31–33]. Future work will be required to investigate and fully appreciate the complexity of the intracellular pathways that lead to brain protection from memory-related phenomena via cAMP/PKA/CREB at the hippocampus, although we have not yet studied signal mechanism. Phillips *et al.* [34] found induced expression of p-CREB in vulnerable hippocampal neurons after global cerebral ischemia. In this study, we observed increased expression of p-CREB in CA1 hippocampal neurons in young and aged WT ischemic mice. However, genetic deletion of the IP receptor in young and aged mice significantly decreased the number of p-CREB positive cells. Taken together, our findings suggest that transient global cerebral ischemia mediates CREB phosphorylation and genetic deletion of PGI2 IP receptor decreases pCREB, which act as a molecular marker of memory processing in the hippocampus for cognitive function [14,35,36].

## 4. Experimental Section

### 4.1. Experimental Animals

This study was performed in accordance with the NIH guidelines for the use of experimental animals. All protocols were approved by the Sanford-Burnham Medical Research Institute Animal Care and Use Committee. Young (2–3 months) and aged (12–15 months) WT and IP receptor knock out (IP KO) C57BL/6 mice were maintained in our barrier facility and genotyped by polymerase chain reaction as described earlier [37].

### 4.2. Experimental Groups

The mice were randomized and sample size was calculated before the start of the experiment. The mice were divided into eight groups: Sham, young and aged, WT and IP KO; ischemia, young and aged, WT and IP KO (*n* = 6–8). The investigators were blinded for surgeries and evaluations for functional and histological outcomes.

### 4.3. Induction of Global Cerebral Ischemia: BCCAO

To induce ischemia, mice were anesthetized with 5% isoflurane and intubated with a small-animal respirator (Harvard, type 845, Harvard Inc., Holliston, MA, USA). A midline incision was made, and then the bilateral common carotid arteries were carefully isolated and occluded by artery clips. After 12 min, the clips were removed to restore cerebral blood flow and the incision was closed. The body temperature was maintained at 37 °C throughout the procedure and recovery with a heating pad. Sham animals received the same surgical procedure except that the carotid arteries were not occluded [38].

### 4.4. T-Maze Spontaneous Alternation

T-maze spontaneous alternation was determined in mice by using a previous method with modifications [39]. Mice were placed on the base of a T maze and were given the choice to explore either the right or left arm of the maze for ten consecutive trials. A choice was assumed to be made when the mouse stepped with all four paws into an arm. At that moment, the gate to that arm was closed and the animal was allowed to explore the arm for 5 s.

### 4.5. Open Field Test

Mouse motoric behavior testing was performed using an open field scoring system, which measures each mouse’s gross locomotor ability inside a plastic tray (8 × 11 × 3 inches) according to a modified Tarlov scale. Locomotor activity scores of mice ranged from 0 (flaccid or spastic paralyis) to 5 (normal walking) as previously described [40].

### 4.6. Immunohistochemistry and Quantification

Seven days after ischemia, paraffin-embedded sections were de waxed and rehydrated. Antigen retrieval was achieved by microwaving sections in citric acid (pH 6) for 5 min. Endogenous peroxidase activity was quenched with exposure to 10% hydrogen peroxide for 30 min. Sections were blocked for 1 h in 4% normal goat serum before being exposed to primary antibody overnight at 4 °C. Anitbodies used were: NeuN (Millipore chemicon, Billerica, MA, USA), specific for neurons or GFAP (Abcam, Cambridge, UK), specific for astrocytes, Iba 1 (Wako Chemicals USA, Richmond, VA, USA), specific for microglia/macrophages and MPO (Dako North America Inc., Carpinteria, CA, USA), specific for neutrophils, anti-phosphorylated-CREB (Ser 133) (Cell signaling, Danvers, MA, USA), specific for pCREB. Following overnight incubation at 4 °C, sections were incubated in appropriate secondary antibody (Vector Laboratories, Burlingame, CA, USA) for 1 h at room temperature. Staining was visualized with the ABC-DAB system (Vector Laboratories, Burlingame, CA, USA). The number of positively immunostained cells in the whole hippocampal CA1 subfield of each section was quantified using the Aperio Scan Scope CS system (Aperio Technologies, Vista, CA, USA). The acquired digital images representing whole tissue sections were analyzed by applying the Spectrum Analysis algorithm package and Image Scope analysis software (version 9; Aperio Technologies) to quantify IHC and histochemical staining.

### 4.7. Statistical Analysis

We used GraphPad PRISM for all statistical tests. We performed a two way ANOVA for behavior tests and cell counts between the young and aged WT and IP KO experimental groups followed by Fisher’s Protected Least Significant Difference (PLSD) post hoc analysis. An alpha level of 0.05 was used to reject the null hypothesis. Data are presented as mean ± standard error of the mean (s.e.m.), and a *p* value < 0.05 was considered statistically significant.

## 5. Conclusions

In summary, this is the first report suggesting that the genetic deletion of the IP receptor exacerbates cognitive and neuronal damage caused by BCCAO in the mouse. Moreover, we found that activated microglia and MPO levels were higher in young and aged ischemic IP KO mice than in WT ischemic controls. Furthermore, we found that p-CREB was decreased in young and aged IP KO mice as compared to their corresponding controls. These results suggest that genetic deletion of the IP receptor exacerbates inflammation due to ischemia, leading to increased neuronal damage accompanied by decreasing expression of p-CREB, which results in the enhancement of memory impairments induced by BCCAO, especially in aged mice.

## References

[1] Peskine A, Picq C, Pradat-Diehl P (2004). Cerebral anoxia and disability. Brain Inj.

[2] Yager J, Andersen AE (2005). Clinical practice. Anorexia nervosa. N Engl J Med.

[3] Ritter L, Funk J, Schenkel L, Tipton A, Downey K, Wilson J, Coull B, McDonagh P (2008). Inflammatory and hemodynamic changes in the cerebral microcirculation of aged rats after global cerebral ischemia and reperfusion. Microcirculation.

[4] Neumar RW (2000). Molecular mechanisms of ischemic neuronal injury. Ann Emerg Med.

[5] Floyd RA, Hensley K (2002). Oxidative stress in brain aging. Implications for therapeutics of neurodegenerative diseases. Neurobiol Aging.

[6] Woodward DF, Jones RL, Narumiya S, International union of basic and clinical pharmacology (2011). LXXXIII: Classification of prostanoid receptors, updating 15 years of progress. Pharmacol Rev.

[7] Kobayashi T, Narumiya S (2002). Prostanoids in health and disease; lessons from receptor-knockout mice. Adv Exp Med Biol.

[8] Dorris SL, Peebles RS (2012). PGI2 as a regulator of inflammatory diseases. Mediators Inflamm.

[9] Anwaar I, Gottsater A, Ohlsson K, Mattiasson I, Lindgarde F (1998). Increasing levels of leukocyte-derived inflammatory mediators in plasma and camp in platelets during follow-up after acute cerebral ischemia. Cerebrovasc Dis.

[10] Masuda Y, Yasuba M, Zushi K, Ochi Y, Kadokawa T, Okegawa T (1988). Effect of OP-2507, a stable prostacyclin analogue on cerebral ischemia induced by unilateral ligation of common carotid artery in gerbils. Arch Int Pharmacodyn Ther.

[11] Gryglewski RJ, Nowak S, Kostka-Trabka E, Kusmiderski J, Dembinska-Kiec A, Bieron K, Basista M, Blaszczyk B (1983). Treatment of ischaemic stroke with prostacyclin. Stroke.

[12] Lundblad C, Grande PO, Bentzer P (2008). Increased cortical cell loss and prolonged hemodynamic depression after traumatic brain injury in mice lacking the IP receptor for prostacyclin. J Cereb Blood Flow Metab.

[13] Saleem S, Shah ZA, Maruyama T, Narumiya S, Dore S (2010). Neuroprotective properties of prostaglandin I2 IP receptor in focal cerebral ischemia. Neuroscience.

[14] Chan EC, Dusting GJ, Guo N, Peshavariya HM, Taylor CJ, Dilley R, Narumiya S, Jiang F (2010). Prostacyclin receptor suppresses cardiac fibrosis: Role of CREB phosphorylation. J Mol Cell Cardiol.

[15] Moncada D, Viola H (2006). Phosphorylation state of creb in the rat hippocampus: A molecular switch between spatial novelty and spatial familiarity?. Neurobiol Learn Mem.

[16] Guzowski JF, McGaugh JL (1997). Antisense oligodeoxynucleotide-mediated disruption of hippocampal camp response element binding protein levels impairs consolidation of memory for water maze training. Proc Natl Acad Sci USA.

[17] Harada N, Taoka Y, Okajima K (2006). Role of prostacyclin in the development of compression trauma-induced spinal cord injury in rats. J Neurotrauma.

[18] Sato Y, Kitani K, Kanai S, Nokubo M, Ohta M (1991). Differences in tolerance to hypoxia/anoxia in mice of different ages. Res Commun Chem Pathol Pharmacol.

[19] Hoth KF, Poppas A, Moser DJ, Paul RH, Cohen RA (2008). Cardiac dysfunction and cognition in older adults with heart failure. Cogn Behav Neurol.

[20] Neumann JT, Cohan CH, Dave KR, Wright CB, Perez-Pinzon MA (2012). Global cerebral ischemia: Synaptic and cognitive dysfunction. Curr Drug Targets.

[21] Danton GH, Dietrich WD (2003). Inflammatory mechanisms after ischemia and stroke. J Neuropathol Exp Neurol.

[22] Kreutzberg GW (1996). Microglia: A sensor for pathological events in the CNS. Trends Neurosci.

[23] Xiao F (2002). Bench to bedside: Brain edema and cerebral resuscitation: The present and future. Acad Emerg Med.

[24] Breckwoldt MO, Chen JW, Stangenberg L, Aikawa E, Rodriguez E, Qiu S, Moskowitz MA, Weissleder R (2008). Tracking the inflammatory response in stroke *in vivo* by sensing the enzyme myeloperoxidase. Proc Natl Acad Sci USA.

[25] Jang S, Dilger RN, Johnson RW (2010). Luteolin inhibits microglia and alters hippocampal-dependent spatial working memory in aged mice. J Nutr.

[26] McGettigan P, Henry D (2000). Current problems with non-specific cox inhibitors. Curr Pharm Des.

[27] Garcia Rodriguez LA, Egan K, FitzGerald GA (2007). Traditional nonsteroidal anti-inflammatory drugs and postmenopausal hormone therapy: A drug-drug interaction?. PLoS Med.

[28] Lonze BE, Ginty DD (2002). Function and regulation of CREB family transcription factors in the nervous system. Neuron.

[29] Montminy MR, Gonzalez GA, Yamamoto KK (1990). Regulation of cAMP-inducible genes by CREB. Trends Neurosci.

[30] Gonzalez GA, Montminy MR (1989). Cyclic AMP stimulates somatostatin gene transcription by phosphorylation of CREB at serine 133. Cell.

[31] Yiu AP, Rashid AJ, Josselyn SA (2011). Increasing CREB function in the CA1 region of dorsal hippocampus rescues the spatial memory deficits in a mouse model of Alzheimer’s disease. Neuropsychopharmacology.

[32] Mizuno M, Yamada K, Maekawa N, Saito K, Seishima M, Nabeshima T (2002). CREB phosphorylation as a molecular marker of memory processing in the hippocampus for spatial learning. Behav Brain Res.

[33] Zhou Y, Won J, Karlsson MG, Zhou M, Rogerson T, Balaji J, Neve R, Poirazi P, Silva AJ (2009). CREB regulates excitability and the allocation of memory to subsets of neurons in the amygdala. Nat Neurosci.

[34] Yamamoto Y, Shioda N, Han F, Moriguchi S, Nakajima A, Yokosuka A, Mimaki Y, Sashida Y, Yamakuni T, Ohizumi Y (2009). Nobiletin improves brain ischemia-induced learning and memory deficits through stimulation of camkii and CREB phosphorylation. Brain Res.

[35] Chen Y, Huang X, Zhang YW, Rockenstein E, Bu G, Golde TE, Masliah E, Xu H (2012). Alzheimer’s beta-secretase (BACE1) regulates the cAMP/PKA/CREB pathway independently of beta-amyloid. J Neurosci.

[36] Kim DH, Jeon SJ, Son KH, Jung JW, Lee S, Yoon BH, Choi JW, Cheong JH, Ko KH, Ryu JH (2006). Effect of the flavonoid, oroxylin a, on transient cerebral hypoperfusion-induced memory impairment in mice. Pharmacol Biochem Behav.

[37] Kobayashi T, Tahara Y, Matsumoto M, Iguchi M, Sano H, Murayama T, Arai H, Oida H, Yurugi-Kobayashi T, Yamashita JK (2004). Roles of thromboxane A(2) and prostacyclin in the development of atherosclerosis in apoE-deficient mice. J Clin Invest.

[38] Olsson T, Wieloch T, Smith ML (2003). Brain damage in a mouse model of global cerebral ischemia. Effect of NMDA receptor blockade. Brain Res.

[39] Matchett GA, Calinisan JB, Matchett GC, Martin RD, Zhang JH (2007). The effect of granulocyte-colony stimulating factor in global cerebral ischemia in rats. Brain Res.

[40] Krajewska M, You Z, Rong J, Kress C, Huang X, Yang J, Kyoda T, Leyva R, Banares S, Hu Y (2011). Neuronal deletion of caspase 8 protects against brain injury in mouse models of controlled cortical impact and kainic acid-induced excitotoxicity. PLoS One.

