# Correction: Shakil, H.; Saleem, S. Genetic Deletion of Prostacyclin IP Receptor Exacerbates Transient Global Cerebral Ischemia in Aging Mice. *Brain Sci.* 2013, *3*, 1095–1108

**DOI:** 10.3390/brainsci11050624

**Published:** 2021-05-13

**Authors:** Hania Shakil, Sofiyan Saleem

**Affiliations:** 1 Hamdard College of Medicine and Dentistry, Hamdard University, Sharae Madinat Al-Hikmah, Karachi 74600, Pakistan; doc_hania@hotmail.com; 2 Center for Neuroscience, Aging and Stem Cell Research, Sanford Burnham Medical Research Institute, La Jolla, CA 92037, USA

The authors wish to make the following corrections to this paper: ref. [1] due to identical images of p-CREB immunostaining for 2–3 months old Ischemia IP KO mice (top right panel) and 12–15 months old Ischemia WT mice (left bottom panel) in **Figure 7A**, replace:

**Figure:**
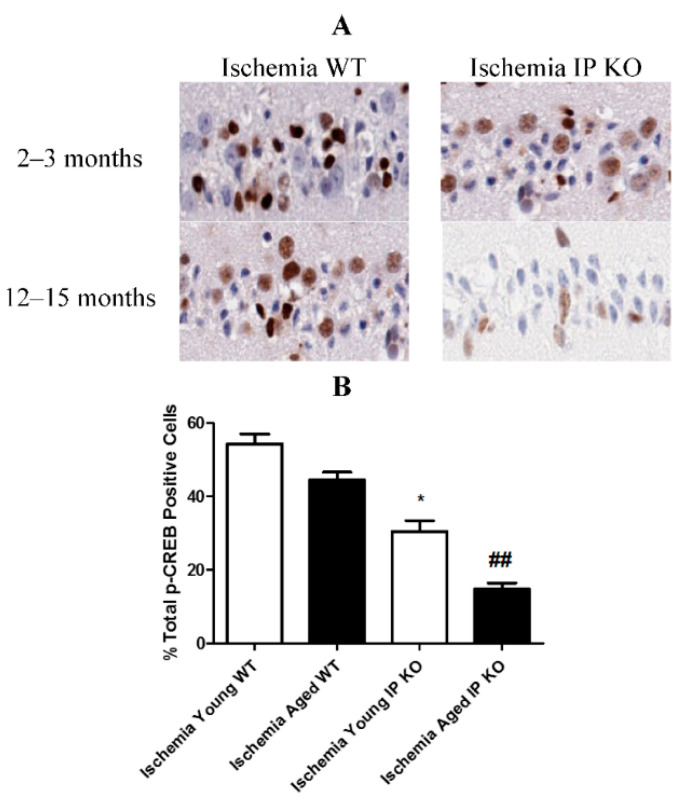


with

**Figure:**
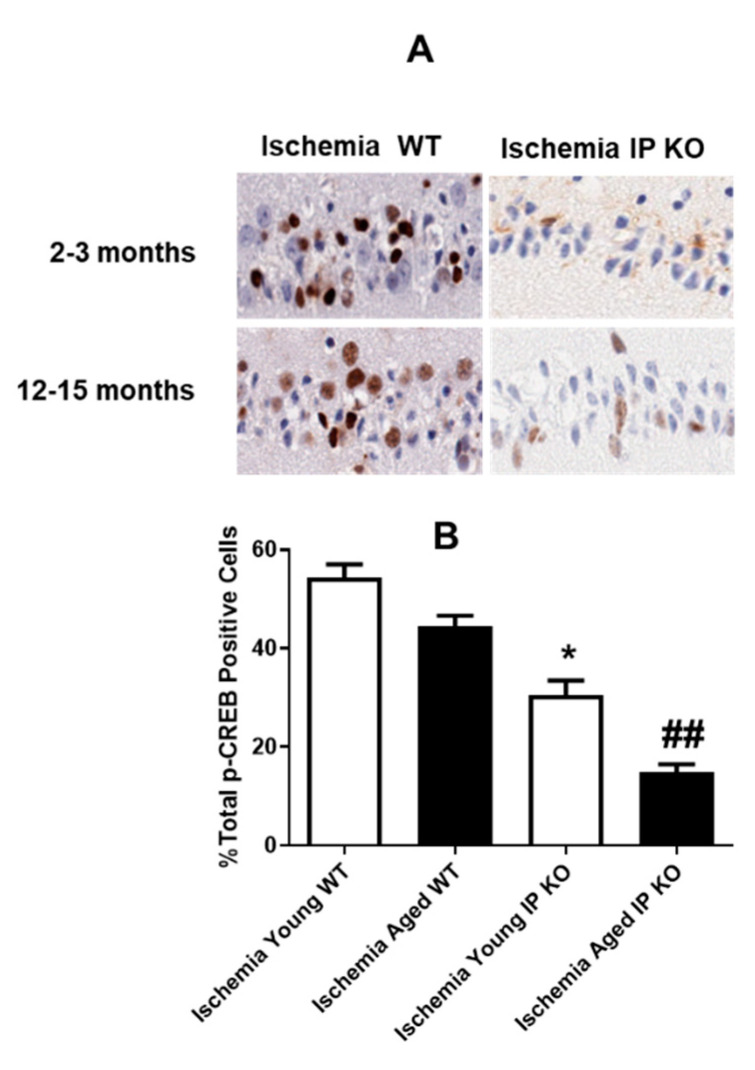


The authors would like to apologize for any inconvenience caused to the readers by these changes.
